# From digital hype to analogue reality: Universal simulation beyond the quantum and exascale eras

**DOI:** 10.1016/j.jocs.2020.101093

**Published:** 2020-10

**Authors:** Peter V. Coveney, Roger R. Highfield

**Affiliations:** a Centre for Computational Science, University College London, Gordon Street, London, WC1H 0AJ, UK; b Institute for Informatics, Science Park 904, University of Amsterdam, 1098 XH, Amsterdam, Netherlands; c Science Museum, Exhibition Road, London, SW7 2DD, UK

**Keywords:** Computer simulation, Digital computing, Analogue computing, Quantum computing, Exascale computing, Machine learning, Artificial intelligence

## Abstract

Many believe that the future of innovation lies in simulation. However, as computers are becoming ever more powerful, so does the hyperbole used to discuss their potential in modelling across a vast range of domains, from subatomic physics to chemistry, climate science, epidemiology, economics and cosmology. As we are about to enter the era of quantum and exascale computing, machine learning and artificial intelligence have entered the field in a significant way. In this article we give a brief history of simulation, discuss how machine learning can be more powerful if underpinned by deeper mechanistic understanding, outline the potential of exascale and quantum computing, highlight the limits of digital computing – classical and quantum – and distinguish rhetoric from reality in assessing the future of modelling and simulation, when we believe analogue computing will play an increasingly important role.

## Highlights

• When machine learning is applied in ignorance of fundamental laws of nature, it is likely to deliver unreliable answers.
• With his colleagues, Peter Coveney has described the computational algorithms best suited for deployment on exascale architectures.
• In the exascale era, we will combine models of the human heart and blood circulation to describe the cardiovascular system at the scale of a body.
• Beyond the quantum and exascale computer eras, the future of simulation will rely more on analogue computing than has hitherto been expected.

## 1 Introduction

We first glimpsed the potential of computers to model the world eight decades ago, in 1936, when Alan Turing devised a hypothetical computing machine while studying the foundations of mathematics [1]. Today, we use computers to describe the turbulence [2] and dynamics [3,4] of air, water and more complex fluids [5], to understand the electronic states of molecules and the kinetics of chemical reactions, for the discovery and understanding of advanced materials, to predict weather and future climate change [6], refugee migration [7], drug design and personalised medical treatments, the creation of virtual organs [8] and, we anticipate, virtual humans too.

In many of these applications, the computer is programmed to solve partial differential equations that are bereft of analytical solutions; and, of course, they can also be used to describe discrete systems, such as lattice gases and other models of fluids, gene regulatory networks with small numbers of molecules [9], agent-based simulations in ecology [10], economics [11] and epidemiology, population dynamics [12], and so on.

Extraordinary progress has been made in recent decades towards the next performance barrier which should be transcended in 2021 with the first exascale computers capable of at least one exaflop [13]. Digital computation is also poised to escape the confines of Moore’s law [14] with the advent of quantum computing [15], leading to feats of modelling and simulation far beyond those achievable by classical computing, or so we are led to believe. A 1000-qubit device would theoretically handle more simultaneous calculations than there are particles in the known universe. It is important not to get carried away, however; today, the biggest quantum computers boast only a few tens of qubits.

Recently, new approaches predicated on machine learning (ML) and artificial intelligence (AI)—terms which are often used interchangeably and synonymously in association with “big data”— have become prominent in tackling a range of complex problems and are sometimes regarded as unbounded in terms of the scope of their domains of application. All this has created the widespread expectation among the general public that we can effortlessly use computers to create virtual worlds across a range of domains, from cosmic associations of galaxies stretching over one hundred million light-years to the mesoscale that is most directly accessible to our senses, and from the molecular machines in our cells down to structures within the heart of an atom and inside the particles that comprise its nucleus.

To simulate different levels of reality, oceans of electrons within myriad microchips, organised within vast numbers of cores that reside inside thousands of nodes in supercomputers are manipulated by billions of tiny switches that flicker on and off billions of times every second. Will this burgeoning virtual environment ever become rich and textured enough to create a faithful replica of our own universe? Some go much further, and even speculate that the cosmos itself arises from interactions between energy and information [16]. Could the universe itself be a quantum computer [17]?

From a consideration of the limits of what is computable, which we discuss further below, we conclude that, although we have come a long way, digital computers – whether classical or quantum – are more restricted in their potential than many realise. Their power resides in their ability to produce vast quantities of numerical data, which lends them an aura of invincibility, yet our article will draw attention to cases where such numerical output can be wrong. Indeed, as we move into the exascale and quantum eras and start to discern what lies beyond them for modelling, there is considerable potential for surpassing the limitations of digital descriptions by falling back on a form of computation which dates back millennia: analogue computing.

## 2 Dirac’s dream

All models and computer simulations rely on theory, representing yet another example of “the unreasonable effectiveness of mathematics”, as Eugene Wigner observed in 1959 [18]. But theory alone is not enough. Three decades earlier, Paul Dirac had remarked that with the general theory of quantum mechanics almost complete, “the underlying physical laws necessary for the mathematical theory of a large part of physics and the whole of chemistry are thus completely known” [19]. However, he added the critical qualification that “the difficulty is only that the exact application of these laws leads to equations much too complicated to be soluble.”

Analytical calculations become intractable relatively quickly, as shown by Henri Poincaré in 1889 with the three-body problem: even the motion of the Sun, Moon and Earth is a non-integrable system, where analytical methods cannot yield an exact solution. Dirac’s dream of, for example, replacing chemistry experiments with theory is rendered stillborn since, when mixing two substances together to trigger a reaction under typical laboratory conditions, chemists typically blend of the order of 10^24^ molecules, give or take a few.

We can express this difficulty in terms of algorithmic ‘complexity’, the amount of time required by a Turing machine to solve a problem with a given algorithm. This is a useful lens through which to view the challenge of modelling. The algorithms used to describe computable problems can be divided into two classes, based on the length of time it takes a Turing machine to find the solution to a problem, as a function of some quantity *N* that measures its size. When problems are polynomial in *N* (that is, an algebraic power of *N*), they are said to be in class P and tractable – the length of time required to crack them does not become unbounded as the size of the problem increases, although for high values of *N*, it can still take an age.

But a class of problem rapidly becomes intractable when the time required to solve it increases in an exponential fashion. These problems, which are not solvable in polynomial time, are said to be in the class NP. Probably the most famous example is the ‘travelling salesman problem’ but there are many other optimisation problems for which no efficient solution algorithm has been found, such as satisfiability and graph-covering problems.

Another way to view the problem of complexity is to consider the extent to which behaviour can be algorithmically 'compressed'. Compression, developed by Andrey Kolmogorov and Gregory Chaitin, expresses complexity as the size of the smallest program which computes it or provides a complete description of it. By this reasoning, if a property of a system is algorithmically incompressible, the most compact way of simulating its behaviour is to observe the process itself.

## 3 Big data need big theory

Even though we do not understand intelligence, and we cannot define it in a scientifically reliable manner [20], “artificial intelligence” has recently returned to the public arena in a prominent manner following a forty-year “AI winter”, notably with the resurgence of artificial neural networks and other forms of ML [21]. In this, the age of so-called “big data”, researchers are increasingly relying on ML to make sense of the world, ironically often without recourse to any form of explanatory insight. Today, there is a pervasive and growing belief that problems of arbitrary complexity are tractable using ML, and in any domain.

The enormous interest in applying ML and AI has proved so intoxicating that some researchers claim that we can dispense with conventional approaches to science, relying on big data to “teach us” how the world works [22,23]. Does this mean the end of theory and the scientific method?

If that were possible, it would eliminate the need for mechanistic, physics based, modelling and simulation, in favour of acquiring and analysing vast amounts of data. This is particularly seductive in areas of biology and medicine, where there remains a substantial lack of fundamental understanding of processes. AI methods should indeed be able to automate diagnostics, notably through pattern recognition [24], and some treatment methods, based on historical data and existing medical treatments [25]. But there is a limit to what can be achieved and, in medicine, regulatory authorities will always require mechanistic explanations before they can approve new drugs and other forms of treatments to make sense of their action and damaging side-effects. On average, each new “once-size-fits-all drug” today costs billions, takes over ten years to bring to market and – at best – works for around 50 % of the population, roughly speaking (see, for example, studies of antidepressants [27], neuraminidase inhibitors [28], and migraine [29]) – most common medications have small-to-medium effects [26] and for reasons that are well known [30]. Yet we live in the post-genomic era, when sequencing an individual’s DNA is accurate and affordable.

Ultimately, one would like doctors to be able to make actionable predictions based on data, such as a genotype, computer models and simulations – by this we mean timely predictions which give doctors the opportunity to improve or cure the condition of a patient, whether in picking one antimicrobial drug in preference to another when confronted with a severe infection, or selecting the best approach for risky life-saving surgery.

Currently, the kind of theory used in biology and medicine tends to offer *post hoc*, i.e. retrospective, explanations of the kind seen in dietary advice and financial markets—for which past performance is no guarantee of future results—rather than scientific ones in their most powerful sense, where predictions are made that, when subsequently tested, are shown to be correct or their failures explained. Indeed, no medical treatment will pass muster with regulatory authorities (such as the FDA in USA and the EMA in Europe) if based simply on the predictions of a ML algorithm. It is essential that an underlying mechanistic explanation is provided.

There is a need for more theory in biology and medicine because no matter the ‘depth’ of neural networks, and the sophistication of data-driven methods, they merely fit curves to existing data. They may be capable of interpolation between data in a training domain provided these relationships are smoothly varying – something which cannot be guaranteed. However, effective extrapolation beyond this domain is fraught because these relationships do not capture the structural characteristics – the underlying mechanism – of the system [22,23]. Complex models commonly overfit data, such that they do not perform well on new compared with existing data [31]. When ML is applied in ignorance of fundamental laws of nature, it is likely to deliver unreliable answers [23].

Even using a rigorous predictive statistical framework—often noticeably lacking from ML studies—characterizing average behaviour from big data will not deliver “personalized medicine” for two reasons. The first is that it is an inference-based approach, so it depends on assigning common properties to “related” data sets, but ultimately every patient is different. The second shortcoming arises from the fact that the amount of data is, and almost certainly always will remain, tiny relative to the complexity of biological systems, which are heterogeneous in space and time. Indeed, the larger the data set acquired, the more problems will beset black box methods such as ML: the ratio of false to true correlations increases rapidly with the size of the data set, making it difficult to ascertain which ones are meaningful and which are not without mechanistic explanations [22]. Sauro Succi and Peter Coveney have explored the many further problems that could arise if and when we have too much data to play with [23].

Today the practice of science is neither strict empiricism (data without reason) nor strict rationalism (reason without data); rather, it is an integration of both with respect to experimental design, predictive modelling and verification. Viewed this way, many big data approaches are pre-Baconian in that they are unconstrained by experimental design and place undue emphasis on blind data gathering without even *post hoc* explanations. Devoid of structural explanations, the correlations they reveal are not necessarily evidence of dependency. To move beyond this pre-Baconian thinking, we need more reliance on theory to produce more reliable predictive models.

Where ML methods are at their most powerful and reliable is when they are used in conjunction with mechanistic understanding, which focuses their use on acquisition of data which is properly targeted to clarify complex but known or relationships anticipated on sound theoretical grounds. Because of issues such as nonlinearity, non-locality and hyperdimensions – which are encountered frequently in multi-scale modelling of complex systems, for example – big data should and will ultimately be used to complement and enhance theory, rather than render it obselete. The field of big data is hugely important but it will not realise its full potential to generate useful knowledge without big theory [22].

## 4 Classical computational chemistry

Some of the greatest challenges to modelling and simulation can be found in chemistry. Despite all the information gathered about hydrogen and oxygen down the years in centuries of experiments, and developments made in the wake of the quantum revolution, we still cannot convincingly predict the boiling point of water from the “bottom up”, that is from the atomic properties of hydrogen and oxygen.

Even so, much important progress has been made over the past half century, for instance harnessing quantum mechanical and Newtonian laws of motion – see below – to examine how drugs bind with target proteins to aid the development of pharmaceuticals. There is an urgent need for accelerated methods of drug development: the traditional model is broken – it costs $2.6 billion to develop a new drug, with gestation times of a decade or longer [32], and no longer fit for purpose in our post-genomic era, where we all know that individuals respond to drugs in different ways because their genomes are different. We need a new kind of precision medicine which at least stratifies the human population into different groups which all respond in known ways to a given drug.

Computer based modelling offers a powerful way to accelerate drug development. Ligand binding models are used to identify the binding sites of receptors but it is well known that tiny changes in the structure of these small organic molecules can sometimes produce very large changes in the strength of binding – so-called “affinity cliffs” [33]. This is one of the reasons why the use of ML remains largely unsuccessful here. To correctly capture such variations using these approaches would necessitate inordinately large quantities of data, which are unlikely ever to be forthcoming [23].

To compute how candidate drug molecules interact with a target protein, ensemble-based methods can be used, where collections of individual Newtonian molecular dynamics are initialised randomly by assigning velocities to all atoms based on the Maxwell-Boltzmann distribution. Because the behaviour of these systems is chaotic, and thus exquisitely sensitive to initial conditions, such ensemble methods reduce the uncertainty of individual “one-off” simulations, making such modelling much more accurate and reproducible [34].

By using an ensemble approach and classical dynamics, the binding affinity, or free energy of binding of a ligand to a protein, can be computed with a high degree of accuracy and precision [35]. Critically, such an approach helps to render the methods reproducible. The results tally with experimental data, which is as important for drug discovery as it is essential to the scientific method; moreover, it makes the calculations actionable. These binding affinities can be calculated using high-performance computing within a few hours, thereby ranking all available drugs in order of their effectiveness for a given person, a development that heralds the development of truly personalized medicine [[36], [37], [38]]. As a consequence, it is now feasible to use simulations to provide timely information about an individual’s mutations responsible for acquired resistance, for example that interfere with the binding of breast cancer drugs (such as Tamoxifen and Raloxifen).

Ensemble-based methods help boost the reproducibility of computational science. When it comes to molecular dynamics simulations, we can run ensembles of 50 different simulations to quantify the uncertainty, for instance when calculating binding free energies. Although this puts yet more demands on the size of computers needed for our models, one can run them concurrently on many of today’s supercomputers and will certainly be able to do so on exascale machines.

Aside from the use of ensemble methods, we need reassurance in other ways if we are to trust our models - we seek confidence that simulation codes are solving the correct governing equations (validation), are being used correctly (verification), with a comprehensive estimate of the uncertainties in the result (uncertainty quantification), where verification, validation and uncertainty quantification are collectively known as VVUQ. However, though this need is widely acknowledged, it is far from being universally implemented, not least because of difficulty obtaining sufficient computing power. One of us (PVC) has helped to develop EasyVVUQ to make state of the art VVUQ algorithms available to a wide range of computational scientists [39]. However, many research findings in computational science are not reproducible for other reasons, such as lack of access to the same data sets and the expense of independently gathering these data [40], so reproducibility will continue to be a challenge.

## 5 Quantum chemistry

Although researchers have long sought methods to model bonds between the atoms in molecules to calculate molecular properties and the interplay between molecules, the applications within chemistry had to wait until the 1960s when computers came into general use for solving these equations and quantum chemistry emerged, marking an important step towards realising Dirac’s dream.

Walter Kohn simplified the mathematics in descriptions of the bonding between atoms, a prerequisite for many of today’s calculations [41]. He showed that it is not necessary to consider the behaviour of individual electrons – it suffices to know the average number located at any one point in space, creating a computationally simpler method called density-functional theory [42]. This approach can, for example, be used to explain how enzymatic reactions occur [43].

In 1998, Kohn shared the Nobel prize with John Pople, who developed the quantum-chemical methodology that facilitated the theoretical study of the behaviour of molecules in chemical reactions, through his GAUSSIAN computer program [44]. At the beginning of the 1990s, Pople was able to incorporate Kohn’s density-functional theory into that code’s algorithms, opening further possibilities for analysing more complex molecules.

Today, quantum chemistry can predict the equilibrium structures of molecules, transition states, intermediates and reaction paths, along with various molecular properties and intermolecular interactions to study solvent effects, crystal packing and so on [45]. However, it is hobbled by the intractability of most of its algorithms. The fastest and most approximate of these algorithms scale as powers of *N* of four, or higher, where *N* means the number of orbitals which are used in the quantum chemical calculation. The greater *N*, the more electrons in the system, meaning the larger the molecule under study, but also the more accurate the calculations for a molecule of given size. The most accurate of these calculations are so-called coupled cluster methods; in their full glory they scale factorially with *N*, which is even more demanding of computing power than those that scale exponentially with the size of the problem. For these reasons, despite the enormous acceleration in the speed of computers over the past several decades, it remains infeasible to calculate the behaviour of large molecules and assembles of molecules of most relevance to real world chemistry, physics, biology and medicine.

However, a glimpse of the solution to this problem came as long ago as 1981, when Feynman delivered his seminal lecture 'Simulating Physics with Computers' [46]. As he put it, somewhat trenchantly: “Nature isn't classical, dammit, and if you want to make a simulation of nature, you'd better make it quantum mechanical.” Many researchers now place their faith in quantum computing, discussed below, to render these calculations tractable within the coming decade or more.

## 6 Multiscale modelling

In the discovery of advanced materials, similar challenges to those seen in drug development present themselves. It can typically take twenty years and many billions of dollars to design a new material for an aerospace component and see it implemented in an aircraft. The quest is still dominated by trial and error, and extensive experimental testing.

When using modelling to predict the behaviour of materials, there is an additional complication to consider. The breaking of a single chemical bond, by electron rearrangement, can cause the fracture of an entire aircraft wing. That is an example of a multiscale process working to devastating effect. The challenge to modelling is how to convolve many scales – from the precise chemical combination of the atoms and their electronic structure to descriptions of matter which are oblivious to the existence of atoms and molecules.

To address a diverse range of problems, from modelling how breaking bonds lead to cracks, and how the movement of ions along molecular pores affect the performance of organs, one has to start at the quantum mechanical level, then lace this description into tiered levels on longer length and time scales, typically including atomic and molecular dynamics, a mesoscale domain and, finally, the macroscopic scale, where matter is represented as a continuum (using finite element methods). Combining these theories to bridge different levels of description is not easy [47]. One reason is that the theories used in each domain (quantum, classical, continuum) are not necessarily compatible - for example some have an arrow of time while others are indifferent to time’s direction [48].

To describe condensed phases of matter rather than isolated molecules in the gas phase, Martin Karplus [49], Michael Levitt and Arieh Warshel in the 1970s developed hybrid methods – density-functional theories – that blend the best of classical and quantum methods [50]. They could draw on the facility of classical physics, where calculations were relatively simple enough to model large molecules, together with quantum insights into chemical reactions. By making Newton’s classical physics work alongside quantum physics they could, for example, simulate how a drug couples to its target protein by performing quantum theoretical calculations on those atoms in the target that interact with the drug, simulating the rest of the protein using less demanding classical physics [51]. Because this methodology has been widely used in organic chemistry and biochemistry, along with heterogeneous catalysis and theoretical calculation of the spectrum of dissolved molecules, they shared the Nobel prize in chemistry in 2013.

There are many more recent examples of multiscale modelling. Studies by one of us (Peter Coveney) have focused on simulating one family of composites in which clays have been combined with synthetic polymers (such as nylon) to produce superior properties [52,53]. We also investigated how graphene influences the properties of epoxy resins [54]. Through our work on the latter problem we seek to reliably design 2D nanocomposite materials through multiscale materials modelling. Reassuringly, quantum simulations of material properties performed by different researchers and with different software are both reproducible and able to produce identical results [55].

These efforts complement those at Los Alamos, where another team is using first principles methods to guide, enhance and refine a variant of the materials genome initiative [56]. They do this by applying statistical methods to direct data collection based on both experimental measurements and electronic density functional theory for the discovery of new materials [57,58]. In the long run, these novel approaches will cut the time to roll out new materials from laboratory to the production line from decades to years, months and, one fervently hopes, days.

ML can complement multiscale modelling, if used judiciously. With the help of ML, it is possible to create robust predictive models [59] such that, where ML reveals a correlation, multiscale modelling can test whether this correlation is causal [60]. If other challenges can be overcome (the creation of robust predictive mechanistic models from sparse data, ways to manage poorly-posed problems and so on) the use of ML to produce less compute-intensive surrogate models will help multiscale modelling to become central to our understanding of complex systems, such as those found in advanced materials, biology and medicine [59].

Multiscale computing is now widespread but, although the field is gaining in maturity, there is a need for further developments to make the most of future exascale and quantum computers. Uncertainty quantification is largely an open subject in this context, but one which may be facilitated by the advent of the exascale.

## 7 Exascale computing

Within the next two years we expect to see the first exascale computers. What sort of challenges will we then be able to address that are currently out of reach?

A glimpse of the bleeding edge of modelling comes from the winners of the ACM Gordon Bell prize [61] for innovations in supercomputing. This year, the prize went to a team from the Swiss Federal Institute of Technology Zurich for “A Data-Centric Approach to Extreme-Scale *Ab Initio* Dissipative Quantum Transport Simulations”. They used Piz Daint at the Swiss National Supercomputing Centre and Summit at Oak Ridge National Laboratory, US, to better understand the thermal properties of transistors which would, appropriately enough, help manage heat generation and dissipation as computer architecture shrinks [62].

Last year, a team affiliated with the Oak Ridge National Laboratory was recognized for their paper “Attacking the Opioid Epidemic: Determining the Epistatic and Pleiotropic Genetic Architectures for Chronic Pain and Opioid Addiction,” along with a second affiliated with the Lawrence Berkeley National Laboratory for “Exascale Deep Learning for Climate Analytics.” Both reported new performance hikes through running their applications on Summit at the Oak Ridge Leadership Computing Facility. In 2017, a simulation carried out on China’s Sunway TaihuLight created 3D visualizations relating to a devastating earthquake that occurred in Tangshan, China in 1976. Although earthquake prediction is an inexact and emerging research area, the use of supercomputers this way may lead to better prediction and preparedness – in this case, to inform engineering standards for buildings in seismically active zones.

Examples of forerunners of exascale machines include Summit, mentioned above, which has an IBM Power 9 architecture with 6 NVIDIA V100 GPUs per node and a peak performance of around 200 petaflops. Another, Fugaku (Japanese for Mount Fuji), the successor to the K supercomputer being developed by RIKEN and Fujitsu in Kobe, Japan, will have ARM AF64FX accelerators and is scheduled to go into service in 2021, while Aurora, an Intel machine with Intel’s own GPUs, should be installed at DoE’s Argonne National Laboratory near Chicago in 2021 and is intended to hit the exascale in the second half of that year.

Exascale machines are unprecedented for reasons other than raw speed. Their architectures will be far more complicated than previous supercomputers, displaying a level of heterogeneity which makes it challenging for us to programme them effectively. That is why the notion of co-design is so important at this scale: programmers need access to the detailed hardware layout of proposed machines in order to work with manufacturers and systemware developers to ensure that exascale machines can be used effectively for scientific research as soon as they roll off the production line.

The increase in speed achieved by these machines will come from massive parallelism, where performance is boosted through the aggregation of the speed of all the cores, accelerators, and nodes in the machine via ultrafast proprietary networks. Data management is an increasing challenge, along with power demand [64]. To curb the incessant increase in power required to run these vast machines, individual cores are not generally increasing in performance; if anything, they are decreasing in clock speeds. To put it colloquially, they are becoming “fatter rather than faster” [63]. While they can accelerate many tasks, others are harder to speed up significantly, not least because transistors are already tightly packed on computing chips. One can liken the performance limitations of modern supercomputers to the limitation on the gestation of a human foetus; it takes nine months and cannot be sped up, though multiple babies can be born concurrently on that time scale across a human population.

Significant thought has been put into determining what forms of computing will be best suited to running on exascale machines. With Alfons Hoekstra, Simon Portegies Zwart, and Bastien Chopard, David Coster, Peter Coveney has laid out a description of the kinds of computational algorithms and “patterns” best suited for deployment on exascale architectures [47]. While there will certainly be scope for a limited number of monolithic applications that can run across the full production partition, there will be relatively few such cases; more generally, we expect to see the further rise of multiscale, multi-physics codes that stitch together numerous single scale models to effectively bridge the time and space scales of the most important scientific and engineering challenges of the day.

One of the more extraordinary efforts set to come into its own in exascale machines is dedicated to the creation of virtual humans, also called digital twins [65]. There are multiscale models of the skeletal and musculoskeletal systems [66]. Efforts are also under way to develop virtual livers [67], lungs [68] and kidneys [69]. In particular, great progress has been made since the 1960s in modelling the heart [70], flows in the aorta [71] and to integrate knowledge at the tissue, cellular, and molecular levels. A team led by one of us (Peter Coveney) is currently simulating the blood circulation from the hips down in Yoon-sun, based on high-resolution (0.1 × 0.1 × 0.2 mm) colour cross-sections from a frozen cadaver of a 26-year-old South Korean woman. The simulation runs on up to one half of SuperMUC-NG in Garching (due to its then systemware, so far it has only been possible to run on half of its 311,000 cores, although the first benchmarks across the entire production partition were acquired in late November 2019). The simulation of 3D macroscopic blood flow on a full human scale is now becoming possible [72].

In the exascale era, it will be possible to combine multiple models, such as the human heart and blood circulation, to describe the cardiovascular system at the scale of a body. There are even greater ambitions. Functioning computer models of a brain or part of a brain, from rodent to human, are now being created c.f. EU Human Brain Project [73], Blue Brain [74] and equivalents elsewhere, e.g. in the USA. Here, however, the hype has sometimes overtaken reality. Some dream of creating computers that can realise artificial forms of consciousness [75], a concept that is even more elusive than that of intelligence. Others speculate about transferring the contents their own brains onto a computer [76], or even that natural intelligence inhabits a computer simulation [77]. Given the limitations of digital computers, discussed later, these ambitions seem far-fetched.

## 8 Quantum computing

New horizons beckon with the rise of quantum computing. It purports to offer the means, at some ill-defined but supposedly not-too-distant future, to perform calculations in milliseconds that would take classical supercomputers the age of the universe to do.

In the early 1980s, extending the work of Turing half a century earlier, Richard Feynman [46], Paul Benioff and Yuri Manin [78] provided the groundwork for quantum computing. In 1985, David Deutsch proposed a universal computer based on quantum physics, which would have calculating powers that Turing’s classical computer (even in theory) could not simulate.

Quantum computing remains a relatively immature discipline and progress is being achieved despite the ‘measurement problem’, that is, the lack of deep understanding of the measurement process: there is no generally accepted theory of how (or whether) wave function collapse occurs and what causes it (it lies outside the agreed theory of quantum mechanics) to convert a probabilistic quantum quantity into a classical certainty. Paradoxically, a great deal of effort is being expended to maintain quantum coherence without knowing how to prevent collapse.

Though it is unclear exactly what problems quantum computers will be most effective at solving most agree, as suggested earlier, that quantum chemistry should be one of its main applications [79]. The central expectation of many in quantum computing is that we will “crack” the computational intractability of electronic structure calculations which we discussed earlier using quantum computers – “the quantum simulation of quantum chemistry”. Various attempts have already been made to use quantum computers (or quantum computer simulators) to solve chemistry problems, such as the calculation of lithium hydride’s and other molecular ground-state energies [80], which suggest that accurate ground-state energy calculations should scale linearly with molecular size rather than exponentially, as in classical computing.

Quantum computers have thus far been used to calculate the ground-state energy for hydrogen [81] and molecules of increasing size, up to water and BeH_2_ [82], and there is reason to think that quantum simulations of classically intractable molecules will be viable. [83]. The dream is that one day researchers will be able to use quantum simulations to help in the design entirely new molecules for use as medical drugs. But for quantum computation to become widely used, algorithmic improvements will be needed [84]. Currently, much hope is being placed on the use of variational quantum eigensolvers, which are hybrid schemes combining quantum and classical computational steps, but the field is still in flux and most effort is of a purely theoretical nature. Work by one of us (Peter Coveney), in collaboration with colleagues at Imperial College London and Tufts University, focuses on how quantum algorithms for quantum chemistry can be optimised so as to maximise the use of available quantum computational resources, which are likely to be limited in the near future [85].

## 9 Coping with chaos

We have already alluded to the care that must be taken with modelling and simulating complex systems that are sensitive to chaos, the subtle mathematical idea that simple equations can generate apparently random behaviour. The field of weather forecasting was one of the first to appreciate the need for caution because of the ‘butterfly effect’, where a butterfly flapping its wings in London can, in principle, cause a subsequent hurricane in the Philippines because the system – in this case the Earth's atmosphere – is so delicate [48]. Models have to be kept on track by correcting them with satellite and other sensor-based data, else they would drift away from the true behaviour of the weather.

There is, however, a more fundamental problem that may stymie progress in simulation because of the digital nature of modern computers, whether classical or quantum. Digital computers often handle four billion rational numbers that range from plus to minus infinity, the so-called ‘single-precision IEEE floating-point numbers’, which refers to a technical standard for floating-point arithmetic established by the Institute of Electrical and Electronics Engineers in the 1950s; they also frequently use double precision floating-point numbers, while half precision has become commonplace of late in the running of machine learning algorithms.

However, it is seldom realised by computer and computational scientists, let alone day to day users of these digital machines, that the way these numbers are distributed is highly nonuniform. There are as many IEEE floating point numbers between 0 and 1 (where there are one billion of them) as there are from 1 to infinity, and as many between 0.25 and 0.5 as between 0.5 and 1.0.

Plainly, digital computers only use a very small subset of the rational numbers; and there are infinitely more irrational than rational numbers, which are ignored by all digital computers. The IEEE floating point numbers are all dyadic fractions, that is numbers whose denominators are powers of two. That is a poor representation even of the rational numbers. Recent work by one of us (Peter Coveney) in collaboration with Bruce Boghosian and Hongyan Wang at Tufts University, demonstrates that there are major errors in the computer based prediction of the behaviour of arguably the simplest of chaotic dynamical systems, the generalised Bernoulli map, regardless of the precision of the floating point numbers used [86]. As such, it is a newly discovered pathology of the IEEE floating point number system. Earlier work also points in the same direction [87]. It is remarkable that the simulation community remains ignorant of these problems in the use of digital computers. These findings strongly suggest that the digital simulation of all chaotic systems, found in models used to predict weather, climate, molecular dynamics, chemical reactions, fusion energy and much more, contain sizeable errors [87]. By the same token, the use of data from these chaotic simulations to train ML algorithms will be subject to learning artefacts. For example, Pathak et al. claim that machine learning can be used for “model-free” prediction of the time evolution of chaotic dynamical systems from data alone [88]. This is illustrated using data generated from the solution of a known chaotic model called the Kuramoto-Sivashinsky equation, suggesting that a low prediction error is obtained for about 8 Lyapunov times. Since this model and the data generated from it is likely to contain significant errors arising from the aforementioned pathology, reproducing such errors with an ML algorithm is not per se compelling evidence that model-free prediction works.

Although the so-called “next generation arithmetic” of John Gustafon, based on entities he calls unums and posits [89], offers more flexibility than the IEEE floating point numbers, it cannot mitigate this problem. Research is now underway by Boghosian and Coveney to find alternative approaches that might render such problems computable on digital computers.

Among possible solutions, one that seems guaranteed to succeed is analogue computing, an older and more powerful idea, able to handle the numerical continuum of reality in a way that digital computers can only approximate. While digital computation has seemingly rendered nearly all forms of analogue computation obsolete since the 1950s, various novel approaches are now under development. For example, an analogue approach to the simulation of quantum chemistry problems that relies on the combination of ultracold atoms in optical lattices and cavity quantum electrodynamics, opens up the possibility of analogue quantum simulations of the electronic structure of molecules [90].

## 10 Universe in a computer

Finally, let’s consider the ultimate simulation – could we, one day, simulate our entire universe in a computer? Many hurdles face this extraordinary and hubristic idea: the probabilistic and non-deterministic nature of quantum theory; our incomplete knowledge of the cosmos; our inability to capture the richness of the universe using digital computers; along with deeper philosophical concerns, such as to how such a computer, being part of the universe, must also simulate itself simulating itself in an infinite recursion. The anthropocentric idea that the universe itself is a computer, which rests on the assumption that it operates in the same way as we solve problems, runs into difficulty because of quantum phenomena and the measurement problem [91].

In the wake of the first claim [15] of ‘quantum supremacy’ one can also expect quantum computing to gain increasing attention. Even so, as we gaze toward the long-term prospects for modelling, many of the basic premises of current computing rest on somewhat shaky foundations. In the medium term, less emphasis should be placed on the world of ones and zeros and more on analogue computers, both quantum and classical.

Important progress will be made by complementing increasingly powerful digital computers with classical and quantum analogue machines. In 1941, Claude Shannon provided a theoretical model, the general-purpose analogue computer (GPAC) [92]. Four decades later, Pour-El & Richards [93] and Lipshitz and Rubel [94] showed that we can solve any ordinary differential equation with this type of device, given appropriate boundary conditions as part of the input. There is also a fully quantum version of analogue computing, known as continuous-variable quantum computing (CVQC), first described by Lloyd and Braunstein [95]. Carver Mead’s concept of neuromorphic computing is alive today [96], particularly within the Human Brain project, albeit only one of the two approaches being pursued along these lines is truly analogue in nature. Metamaterials also show great promise for optical analog computing at micro- and nano-scales, at near light speeds and using little power [97,98,99].

We may encounter new limitations: the results returned from calculations performed on analogue computers could, at the limit, turn out to be dependent on the nature of the matter (polymers, molecules and atoms) of which they are comprised, just as digital computers are limited by the nature of the digits that they manipulate. Even so, it should be pointed out that the most advanced computational device that we know of happens to be analogue: the human brain. Humans are able to correctly assign the truth of Gödelian statements which cannot be divined by digital computers, underpinning Roger Penrose’s famous argument against the ability of digital computers to produce true artificial intelligence [100]. Moreover, as supercomputers get more energy hungry (the peak energy consumption of Summit is around 13 MW) it is humbling to remember that the human brain requires around 20 W to operate.

Beyond the quantum and exascale computer eras, the future of modelling and simulation may well come to rely more on analogue computing than has hitherto been expected.

## Authorship conformation form

All authors have participated in (a) conception and design, or analysis and interpretation of the data; (b) drafting the article or revising it critically for important intellectual content; and (c) approval of the final version.

This manuscript has not been submitted to, nor is under review at, another journal or other publishing venue.

The authors have no affiliation with any organization with a direct or indirect financial interest in the subject matter discussed in the manuscript.

## Declaration of Competing Interest

None.
